# Seasonal Distribution and Diversity of Ground Arthropods in Microhabitats Following a Shrub Plantation Age Sequence in Desertified Steppe

**DOI:** 10.1371/journal.pone.0077962

**Published:** 2013-10-21

**Authors:** Rentao Liu, Fan Zhu, Naiping Song, Xinguo Yang, Yongqing Chai

**Affiliations:** Key Laboratory for Restoration and Reconstruction of Degraded Ecosystem in Northwestern China of Ministry of Education, Ningxia University, Yinchuan, China; Arizona State University, United States of America

## Abstract

In desertified regions, shrub-dominated patches are important microhabitats for ground arthropod assemblages. As shrub age increases, soil, vegetation and microbiological properties can change remarkably and spontaneously across seasons. However, relatively few studies have analyzed how ground arthropods respond to the microhabitats created by shrubs of different plantation ages across seasons. Using 6, 15, 24 and 36 year-old plantations of re-vegetated shrubs (*Caragana koushinskii*) in the desert steppe of northwestern China as a model system, we sampled ground arthropod communities using a pitfall trapping method in the microhabitats under shrubs and in the open areas between shrubs, during the spring, summer and autumn. The total ground arthropod assemblage was dominated by Carabidae, Melolonthidae, Curculionidae, Tenebrionidae and Formicidae that were affected by plantation age, seasonal changes, or the interaction between these factors, with the later two groups also influenced by microhabitat. Overall, a facilitative effect was observed, with more arthropods and a greater diversity found under shrubs as compared to open areas, but this was markedly affected by seasonal changes. There was a high degree of similarity in arthropod assemblages and diversity between microhabitats in summer and autumn. Shrub plantation age significantly influenced the distribution of the most abundant groups, and also the diversity indices of the ground arthropods. However, there was not an overall positive relationship between shrub age and arthropod abundance, richness or diversity index. The influence of plantation age on arthropod communities was also affected by seasonal changes. From spring through summer to autumn, community indices of ground arthropods tended to decline, and a high degree of similarity in these indices (with fluctuation) was observed among different ages of shrub plantation in autumn. Altogether the recovery of arthropod communities was markedly affected by seasonal variability, and they demonstrated distinctive communal fingerprints in different microhabitats for each plantation age stage.

## Introduction

In fragile ecosystems of arid, semi-arid and dry sub-humid climatic areas, desertification, a human-induced land degradation process, is one of the most serious environmental and socioeconomic problems worldwide and a major threat for the sustainability of agriculture and economic development [1]. In these desertified regions, planting indigenous shrubs in areas of shifting sand has been demonstrated to be an effective strategy to restore degraded ecosystems and control desertification [2]. As reported, shrubs, with their root systems and shading crown, can create high nutrient patches in a low-nutrient matrix (i.e. fertile islands) and can modify the nearby environment as well [3]. The spatial heterogeneity (i.e. different microhabitats) created by shrubs is a key factor for understanding the structure and dynamics of local populations, communities and ecosystems [4], which are fundamental issues in conservation, sustainable management and environmental restoration [5].

It has been suggested that shrubs established in desertified regions can act as resource sinks and provide species-specific shelter from temperature/drought extremes for soil/ground arthropods [3,6]. The distinct structure, physiology and phenology of each shrub species can cause differences in their effects as fertile islands, thus affecting the distribution and diversity of animal communities [5,7,8]. Evidence shows that shrubs, in terms of presence or absence and species identity, can have important influences on the distribution and assembly of soil/ground arthropod communities, even across seasons [3,9,10]. The high temporal variability of these environments may interact with the spatial heterogeneity to produce a dynamic mosaic in the distribution of the soil fauna [10,11]. In addition, the existence and scale of heterogeneity created by shrubs can have important effects on animal movement patterns [12]. Few studies, however, have evaluated how shrub age can influence the distribution and diversity of soil/ground arthropod communities in different microhabitats across seasons in a desertified region, despite the fact that ground arthropods represent a diverse and abundant component of the soil biota and perform important roles in the soil ecology of these nutrient limited desert soils [3,13].

Recently, a great number of studies have explored the effects of shrub age on soil, plant and microbiological properties [14–16]. The effect of the crown changes with shrub age [17]; for example, the physical protection offered by the crown and the presence of soil organic matter and nutrients improve with time [15,18]. Plant species diversity increases as artificial plantation age increases, with increased vegetation height and cover, even across seasons [19,20]. As crowns age, gradients in biomass production and distinct patterns in species distributions appear in the understory, where stress-tolerant species show up towards the edge of the projected crown area while more mesic species stay well inside the crown [21]. As shrub age increases, the ‘island of fertility’ also expands into the open areas between shrubs [14]. These findings indicate that the initially simple, artificial vegetation system develops into a complex ecosystem composted of artificial shrubs and natural herbs, capable of reversing desertification following shrub growth [14,18]. Together with soil seed bank composition and abundance, shrub age has been directly related to the distribution of soil/ground arthropods in arid and semiarid ecosystems [19,21]. However, the influence of microhabitat differences among shrub plantations of different ages on ground arthropods remains unclear across seasons in desertified steppe.

Ground arthropods and their diversity are of considerable significance during the recovery process of degraded ecosystems [22,23], since variations in diversity are presumably correlated with the stability of various biotic and abiotic components of ecosystems [24,25]. Soil biodiversity has become an important measure for the evaluation of ecosystems [24], though the role of species diversity in ecosystem function is disputed [23,26]. Additionally, soil invertebrates have been suggested to serve as indicators of “soil quality” in agricultural and degradation/recovery contexts [27,28]. Therefore, documenting the ecological implications of shrub age on soil arthropod communities in the different microhabitats created by shrubs is essential to develop a valuable management strategy for arthropod diversity conservation and degraded ecosystem recovery [23,24].

A chronosequence of 6-, 15-, 24-, and 36-year-old *Caragana koushinskii* plantations was studied as a model system; these plantations occur widely in the desert steppe of the Ningxia Hui autonomous region (northwestern China) [18]. Using pitfall traps, we investigated the composition and diversity of ground arthropods under the shrub crown (hereafter the ‘under shrubs’ microhabitats) and in the open bare areas between shrubs (hereafter the ‘in the open’ microhabitats) during the spring, summer and autumn, which corresponded to the main period of ground arthropod activity [11]. The main aim of this research was to determine how shrub plantation age influences the distribution and diversity of ground arthropod communities between microhabitats and whether the responses of ground arthropods were consistent across the seasons. We predicted (1) differences in arthropod assemblages between microhabitats across plantation ages; (2) a positive relationship between ground arthropod abundance and diversity and shrub age as soil and vegetation improved with time; (3) a strong influence of seasonality on the distribution and diversity of ground arthropods between microhabitats with plantation age, as a result of variation in rainfall and temperature across the seasons. Information on these changes with plantation age is required for a better understanding of restoration mechanisms and the interactions between soil/ground arthropods and environmental factors. Determining the influence of shrub plantation age will help resource managers formulate a rational invertebrate conservation plan aimed at maintaining ecosystem health and function in the desertified regions.

## Materials and Methods

### Ethics Statement

A Scientific Research and Collecting permit was obtained from the Science and Technology Department of Yanchi County, Ningxia for the study in 2011. No specific permits were required for the described field study. Our study did not involve endangered or protected species. Voucher specimens were deposited in the Key Laboratory for Restoration and Reconstruction of Degraded Ecosystems in Northwestern China of Ministry of Education.

### Study Area

The study was conducted in Yanchi county (37°04´–38°10´N and 106°30´-107°41´E), which is located at the southwest fringe of Mu Us sandy land, in the Ningxia Hui autonomous region, China. This area is a typical agro-pasture transition zone comprising a transition from typical steppe to desert steppe and sandy grassland. The elevation ranges from 1295 to 1951 meters above sea level (1600 meters on average) from North to South, and the region has a temperate continental semi-arid monsoonal climate. Mean annual precipitation is 292 mm, with about 70% of the total precipitation occurring from June to September. Mean annual potential pan-evaporation is 2710 mm. Mean annual temperature is 7.5 °C, and the lowest and highest monthly mean temperatures are -8.7°C in January and 22.4°C in July, respectively. Mean annual wind velocity is 2.8 m s^-1^ and prevailing winds are mainly northwesterlies in April and May. Sand dust blowing at wind velocities over 5.0 m s^-1^ occurs 323 times per year, on average. Wind erosion often occurs from April to mid-June before the rainy season arrives (climate data from Yanchi Meteorological Station, 1976 to 2010). The main soil types are sierozem, loess and orthi-sandic entisols, all of which are poor in fertility, have loose structure and are very susceptible to wind erosion [29,30].

Over the past several decades, the study region has undergone severe desertification and is now a key area for desertification research in China [29]. Desertification started in the 1950s and reached a peak in the late 1970s, caused by disturbance of the stable sandy grasslands from extensive fuel wood gathering and overgrazing [30]. During the desertification peak, shifting, semi-shifting and semi-fixed sand sheets together made up 33% of the total land area. To curb desertification and alleviate its detrimental effects, the local government implemented a conservation program in the 1970s. As part of the program, several native species that are adapted to sandy grasslands were planted, including shrubs such as *Caragana koushinskii*, *Caragana intermedia*, *Caragana korshinskii*, *Hedysarum scoparium* and *Artemisia ordosica*, and forbs such as *Medicago* *spp.*, *Agropyron* *spp.* and *Radix glycyrrhizae*. After fifty years of effort, the percentage of severely desertified land dominated by shifting sand lands in Yanchi county dropped by 64.6% from 1989 (peak desertification) to 2003 [30]. Caragana plantations, both artificial and natural, accounted for an area of *ca*. 24.3% of the county and had a mean density of 915-3015 plant hm^-2^ [31].

### *C*. *koushinskii* Plantations and Management

*C. koushinskii*, a leguminous shrub, is widely distributed in Yanchi county and is a favored plant for restoring vegetation on desertified sandy grasslands [31]. This shrub has been planted around the county, as part of the desertification remediation program that began in the 1970s, with the help of straw checkerboards as sand binders. Planting was arranged in belts (row spacing of 5-7 m and spacing of 1 m) and belt orientation was perpendicular to the prevailing wind direction. Before planting, the dominant plant species in the desertified areas was *Agriophyllum squarrosum*; the vegetative cover was generally less than 6%, and wind erosion often occurred during the dry winter and spring seasons; the soils were very sandy (more than 90% sand) with low organic matter content (0.7 g kg^-1^ organic C content). *C. koushinskii* plants grew to heights of 0.5 m 5-6 years after planting, and formed more continuous belts. With gradual stabilization of the sandy land, some short grasses, legumes and forbs invaded, and a stabilized shrub-grass vegetation system was established. To date, an age series of 6-, 15-, 24-, and 36-year-old *C*. *koushinskii* plantations can be found across the sandy grasslands throughout the county. The 15 and 24-year-old plantations were subjected to livestock grazing prior to 2006, with occasional heavy (1.5 sheep hm^-2^) grazing [29].

### Experimental Design

A common approach in studies of soil rehabilitation in relation to vegetative cover is to monitor plant and soil changes occurring along a vegetative chronosequence developed on similar soils under similar climatic conditions [32]. This chronological approach has been widely used in applied ecosystem research and is considered ‘retrospective’ research because existing conditions are compared with known original conditions and treatments [14].

The retrospective approach was adopted in this study because of the availability of closely located *C*. *koushinskii* plantations established 6-, 15-, 24-, and 36-years ago on sandy soils with similar properties. The plantations, therefore, provide a time series of differing lengths of shrub occupancy on similar sites. Changes in soil arthropod diversity and in environmental factors were measured by comparing sites of different ages. Within each plantation age (6, 15, 24, 36 years), three 10 ha areas were selected 200 m apart from each other. It was assumed that the soils of each site were similar prior to planting shrubs. Recent studies have shown that soil texture (sand, silt and clay contents) and organic matter content do not change significantly in unstable sandy lands over time [18,20], suggesting soils were relatively similar in these characteristics before planting.

At the centre of each sampling site, four *C*. *koushinskii* belts were examined. There were two sampling locations in each belt: under the shrub crown (i.e. under shrubs) and in the open area between belts (i.e. in the open). The distance between two shrub belts that were sampled within a site was >15 m (one every other belt) in order to ensure trap independence [33].

### Ground Arthropod Sampling

Two pitfall traps (7 cm in diameter, 10 cm in depth) filled with approximately 70 ml of 70% ethanol solution were buried, with the opening of the trap level with the ground surface at each sampling belt (one under the shrubs and one in the open area). We thus had eight traps for each site (replicates), 24 traps for each plantation age, and a total of 96 traps across all ages of plantations (Two traps × four belts × three replicate sites × four plantation ages = 96 traps each time). Arthropod sampling was conducted in the spring (5–19 May), summer (11–25 July) and autumn (10–24 September) of 2011; these three seasons collectively represent the main period of ground arthropod activity in our study system [11,20]. Each of the three sampling periods consisted of 15 consecutive days (day and night). Traps were checked every three days during each sampling period and fresh ethanol solution was added when needed. Specimens were preserved in 75% ethanol and identified to family and order level using relevant literature [34,35].

### Measurements of Plant and Soil Properties

For each sampling site, shrub height, crown diameter and aboveground biomass have been previously reported (Figure S1) [20]. Similarly, properties of herbaceous vegetation (plant density, richness, cover and height) and soil (temperature, moisture, bulk density, pH, electrical conductivity and organic carbon and total nitrogen content) have been described elsewhere (Figure S2) [18]. Values of variables from these sources, such as soil texture (sand and clay plus silt content), bulk density, pH, organic carbon content and total nitrogen content were used to explore the relative contributions of different environmental factors to arthropod composition in the current study.

### Data Analysis

Within each sampling site, trap contents were pooled by microhabitat (4 traps within belts and 4 traps between belts). To simplify the analysis, those taxonomic groups with less than three individuals per plantation age were omitted. We then calculated total abundance (number of individuals per 4 traps), total taxonomic richness (the total number of taxonomic groups recorded in each microhabitat per site), and Shannon’s diversity index [9,11,24].

Because of the strong microhabitat and seasonal variations in the ground arthropod assemblages, we have included microhabitat and sampling period as explanatory factors in all the linear models to test the interaction of microhabitat and temporal variability with shrub plantation ages [3,10]. We used general linear model analyses (GLMs, Univariate Analysis of Variance) to compare differences in total abundance, group richness and Shannon index as well as the abundance of some important groups among microhabitats, plantation ages and sampling periods (as fixed factors). We used Tukey-Kramer HSD comparisons to test differences between means for each class within each factor [10]. All statistical analyses were performed using SPSS 16.0 for Windows (SPSS Inc., Chicago, Illinois). Before applying parametric tests, we tested for normality and homogeneity of variances. For all tests, statistically significant differences were assigned at *p*<0.05.

Ordination techniques were used to determine the relative contribution of the measured environmental variables to the community composition [36]. The group abundance data were first analysed by detrended correspondence analysis (DCA). The length of the first DCA ordination axis was 1.283 (for taxonomic group data), suggesting that redundancy analysis (RDA) was an appropriate approach (length of gradient<4) [36,37]. Thus, we used RDA to correlate each arthropod group with the environmental variables (i.e. soil texture [sand and clay plus silt content], bulk density, pH, organic carbon and total nitrogen content), by selecting the linear combinations of environmental variables that gave the smallest residual sum of squares. DCA and RDA were carried out using CANOCO software for Windows 4.5 (Microcomputer Power, Ithaca, USA). Before RDA, a Hellinger transformation [38] was applied to remove the issue of double-zeros in the data matrix and improve the analysis. The data and Monte Carlo reduced model tests with 499 unrestricted permutations were used to statistically evaluate the significance of the first canonical axis and of all canonical axes combined [37]. To meet the requirements of the Monte Carlo reduced model test, all taxonomic group data were square root-transformed.

## Results

### Taxonomic Composition and Distribution of Their Abundances

**Figure 1:**
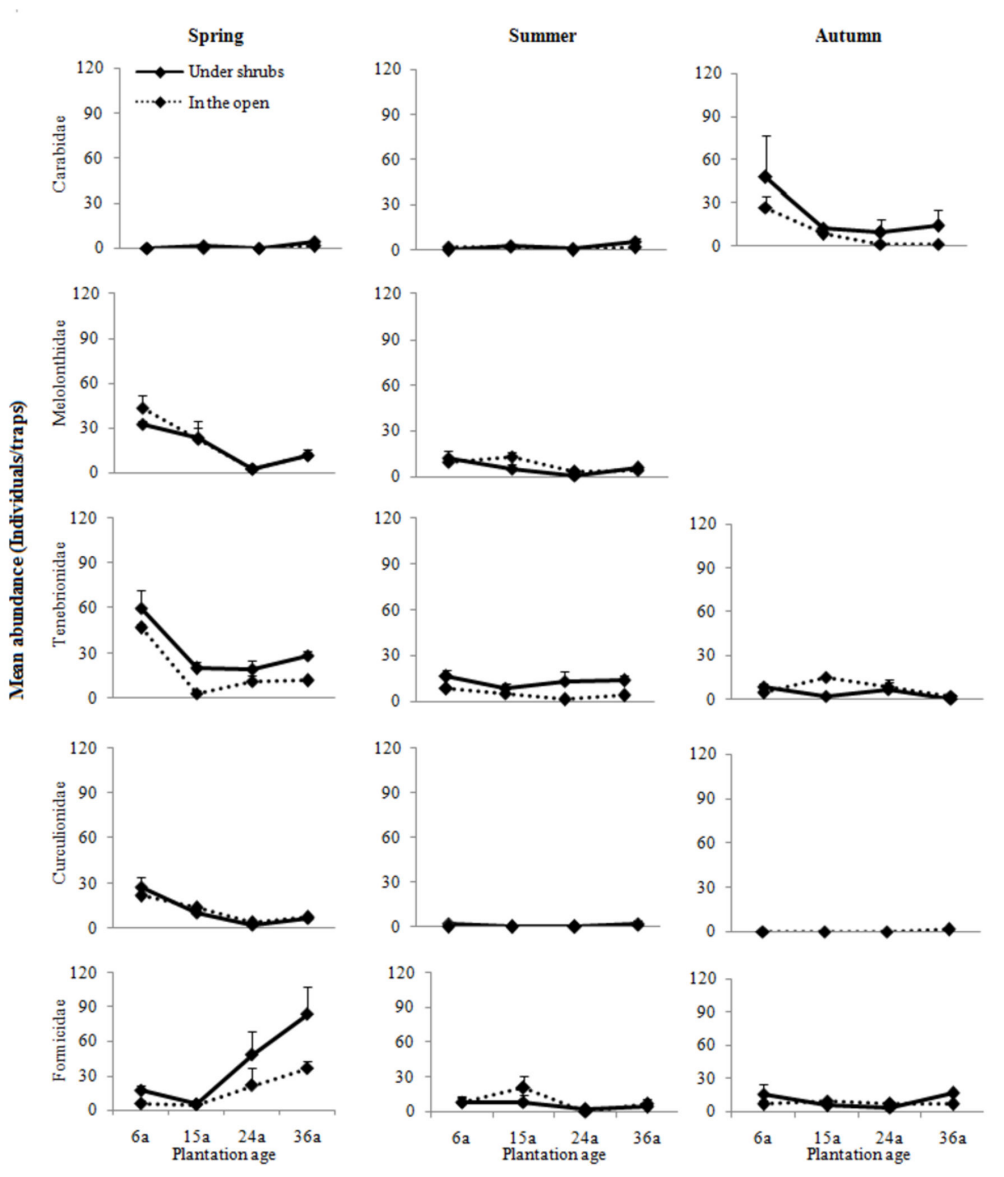
Mean abundances (±SEs) of dominant groups of ground arthropods between microhabitats for each plantation age in different sampling periods.

A total of 3870 individuals belonging to 32 taxonomic groups (10 orders and 30 families plus a larval Lepidoptera) were collected (Table S1). The overall ground arthropod assemblage was dominated by Coleoptera (i.e. Carabidae, Melolonthidae, Tenebrionidae, and subdominant Curculionidae) and Hymenoptera (i.e. Formicidae), which together comprised 86.12% of the total number of individuals (Figure 1).

**Figure 2:**
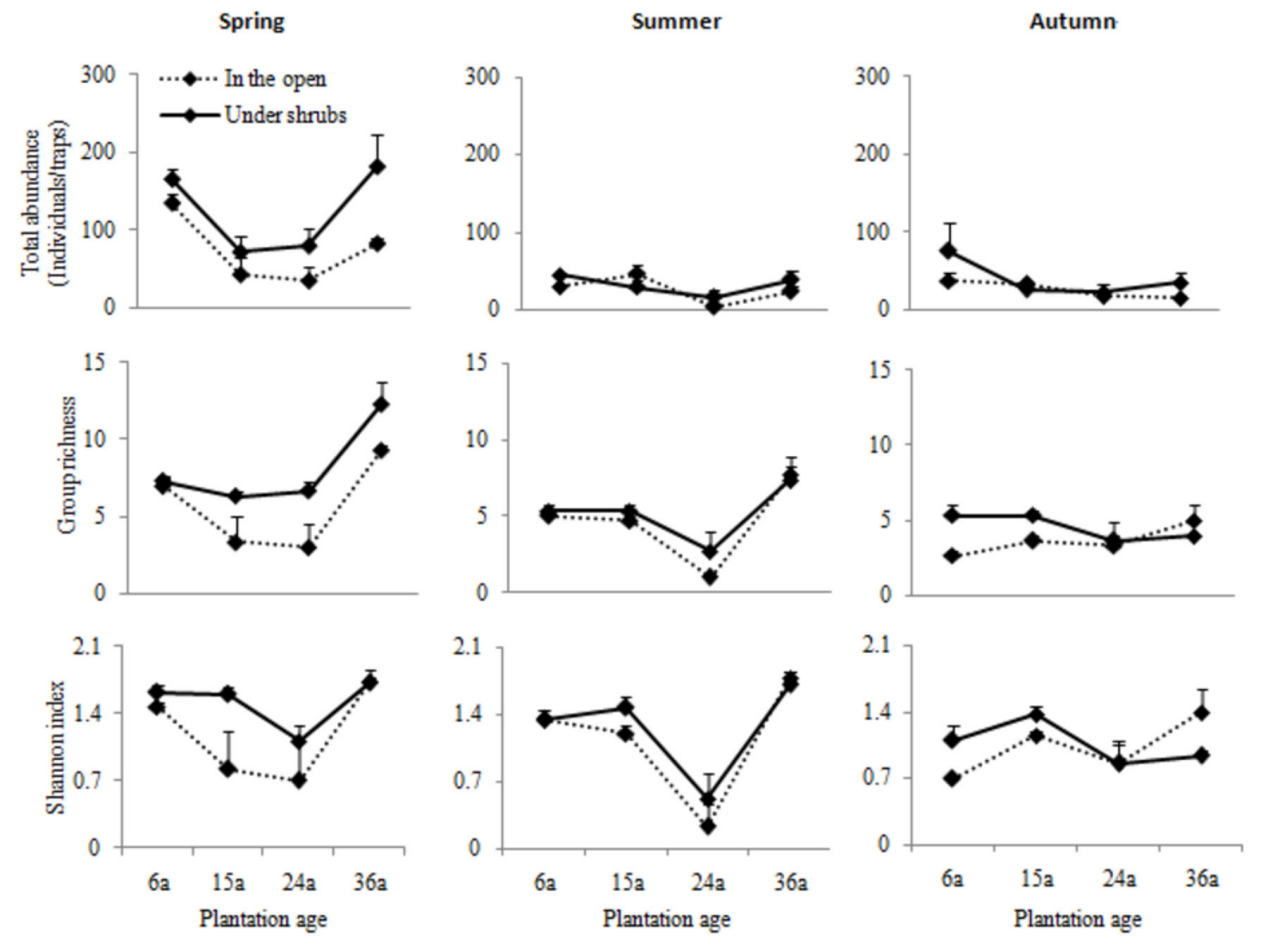
Means (±SEs) of total abundance, taxonomic richness, and Shannon’s diversity index of ground arthropods between microhabitats for each plantation age in different sampling periods.

The total abundance of ground arthropods showed significant differences between the microhabitats under the shrub and in the open areas (Table 1). Total abundance differed also among plantation ages and among sampling periods. There was a significant interaction between microhabitats and sampling periods and between plantation ages and sampling periods for total abundance, indicating that both microhabitat and plantation age variability in total abundance varied among sampling periods. In spring, there was a considerably higher total abundance in the microhabitats under shrubs than in the open areas, and the differences between the two microhabitats tended to increase with plantation ages; from 6 to 24 years of shrub plantation, the total abundance tended to decline, though after this age range they tended to increase (Figure 2). In summer and autumn, on the other hand, there were only slight differences between the microhabitats in the four plantation ages.

**Table 1:** Summary of the general linear models used to test the effects of microhabitat (M), shrub age (A), sampling period (S) and their interaction (M × A, M × S, A × S, M × A × S) on the abundance of dominant groups, total abundance, taxonomic richness, and the Shannon diversity index of ground arthropods.

	*d_f_*	*F*
Carabidae		
M	1	2.67
A	3	2.81*
S	2	10.54***
M×A	3	0.17
M×S	2	1.53
A×S	6	3.43**
M×A×S	6	0.24
Error	48	
Melolonthidae		
M	1	0.67
A	3	17.70***
S	2	52.30***
M×A	3	0.23
M×S	2	0.19
A×S	6	8.94***
M×A×S	6	0.91
Error	48	
Tenebrionidae		
M	1	16.87***
A	3	20.28***
S	2	51.80***
M×A	3	0.72
M×S	2	10.46***
A×S	6	15.45***
M×A×S	6	0.71
Error	48	
Curculionidae		
M	1	0.56
A	3	12.89***
S	2	57.47***
M×A	3	0.54
M×S	2	0.39
A×S	6	12.08***
M×A×S	6	0.25
Error	48	
Formicidae		
M	1	4.62*
A	3	5.20**
S	2	15.75***
M×A	3	2.14
M×S	2	5.25**
A×S	6	7.42***
M×A×S	6	0.88
Error	48	
Total abundance		
M	1	13.46***
A	3	12.50***
S	2	48.60***
M×A	3	1.87
M×S	2	4.71**
A×S	6	4.87***
M×A×S	6	0.73
Error	48	
Group richness		
M	1	15.67***
A	3	27.29***
S	2	24.54***
M×A	3	0.85
M×S	2	3.08*
A×S	6	6.44***
M×A×S	6	1.57
Error	48	
Shannon index		
M	1	5.20*
A	3	22.83***
S	2	5.74**
M×A	3	2.94*
M×S	2	1.48
A×S	6	4.54***
M×A×S	6	0.79
Error	48	

Bold values indicate significant terms (*p = 0.05, **p = 0.01, and ***p = 0.001).

Significant differences in abundance between microhabitats were also observed in two of the dominant taxonomic groups: Tenebrionidae and Formicidae (Table 1). Again, the interaction between microhabitat and sampling period was significant for Tenebrionidae and Formicidae, indicating that the pattern of microhabitat distribution differed among seasons for these two groups. The abundance of all dominant groups varied among plantation ages and among sampling periods. The interaction between plantation ages and sampling period was also significant for all these groups, indicating that the pattern of plantation age distribution differed among seasons for all groups. However, the interaction between microhabitat, plantation age and season was not significant for any groups.

Since the responses of all dominant groups to microhabitats and plantation ages differed across seasons (Figure 1), three different categories were established based on their responses to seasonal variability. The first category included the Carabidae and the Curculionidae, with the former exhibiting the highest abundance in autumn and the latter in spring, although both groups were also present at lower abundances in the other two seasons. In autumn, the Carabidae showed a higher abundance under shrubs than in the open areas in the four plantation ages; in spring, the Curculionidae had a similar abundance distribution between microhabitats in the four plantation ages. The second category included the Melolonthidae only in spring and summer, especially in spring. Again, the abundance distribution of this group was similar between microhabitats in the four plantation ages, and no interaction between microhabitat and plantation age was observed (Figure 1, Table 1).

The third category included the Tenebrionidae and the Formicidae, both being most abundant in spring, though still present in summer and autumn. In spring, there was a higher abundance under shrubs than in the open areas in the four plantation ages for both groups. Overall, no interaction between microhabitat and plantation age was observed for all dominant groups, though plantation age showed marked influences (Figure 1, Table 1). From 6 to 24 years of plantation, the abundances of these groups tended to decline, and after that they tended to increase (except for Formicidae). From 6 to 15 years of plantation, there was a slight change in the abundance of Formicidae, but after that the abundance tended to increase strongly in both microhabitats.

### Group Richness and Diversity Index of Ground Arthropods

Measurements of both group richness and Shannon’s index showed significant differences between microhabitats, and varied also among plantation ages and among seasons (Table 1). Again, the interactions between microhabitat and sampling period and between plantation age and sampling period were significant for group richness (Table 1), indicating that the influence of microhabitat and plantation age also differed among seasons. The interactions between microhabitat and plantation age and between plantation age and sampling period were also significant for Shannon’s index (Table 1), indicating that the influences of microhabitat and also seasons both differed among plantation ages. However, the interaction between microhabitat, plantation age and season was not significant for group richness and Shannon’s index.

In spring, group richness and Shannon’s index were higher in the microhabitats under shrubs than in the open areas (Figure 2); from 6 to 24 years of plantation age, these two indices tended to decline, but after that they increased strongly, though the differences between microhabitats tended to increase slightly with plantation ages (except Shannon’s index in 36 year-old plantations). From spring to summer, group richness and Shannon’s index tended to be similar between microhabitats regardless of plantation age; as plantation ages increased, 24 year-old plantations showed the lowest group richness and Shannon’s index while the highest values corresponded to 36 year–old plantations. From summer to autumn, group richness and Shannon’s index were similar between microhabitats and fluctuated with plantation age. In each season, group richness and Shannon’s index showed similar tendencies as plantation ages increased. Seasonality showed marked influences on group richness and Shannon’s index (Figure 2; Table 1) that declined from spring through summer to autumn.

### Relative Contributions of Environmental Factors to Arthropod Community Composition

Redundancy analysis (RDA) showed that the six environmental variables (Soil sand and clay plus silt content, pH, bulk density, organic carbon and total nitrogen content) together explained 31.6% of the total variation in the data, with axes 1 and 2 explaining 18% and 7% of the total variation, respectively (Table 2). The species-environment relationship for axes 1 and 2 for environmental variables accounted for 79.3% of the total variance, indicating that together, these axes accounted for the bulk of the variance in the arthropod group data that could be attributed to environmental factors. Species-environment correlations for these axes were > 0.81 (Axis 1), indicating that the arthropod group data were strongly correlated with the environmental parameters. Monte-Carlo significance tests revealed that both the first axis (*p*=0.042) and all axes (*p*=0.032) combined explained a significant amount of the variation within the data.

**Table 2:** Results of the redundancy analysis (RDA).

Axis	1	2
Eigenvalues	0.180	0.070
Cumulative percentage variance:		
of species data	18.0	25.1
of species-environment relation	57.1	79.3
Summary of Monte Carlo test:		For all axes:
*F*-ratio	3.96	1.66
*p*-value	0.042	0.032
Species-environment correlations	0.819	0.606
Correlations (*r*):		
SC	-0.646*	-0.102
CS	0.646*	0.102
BD	-0.431	0.326
pH	-0.770*	0.116
SOC	0.584	-0.272
TN	0.572	-0.305
Coefficients (*c*):		
SC	-0.789*	-0.168
CS	0.789*	0.168
BD	-0.526	0.538
pH	-0.940*	0.191
SOC	0.713	-0.449
TN	0.698	-0.503

**Figure 3:**
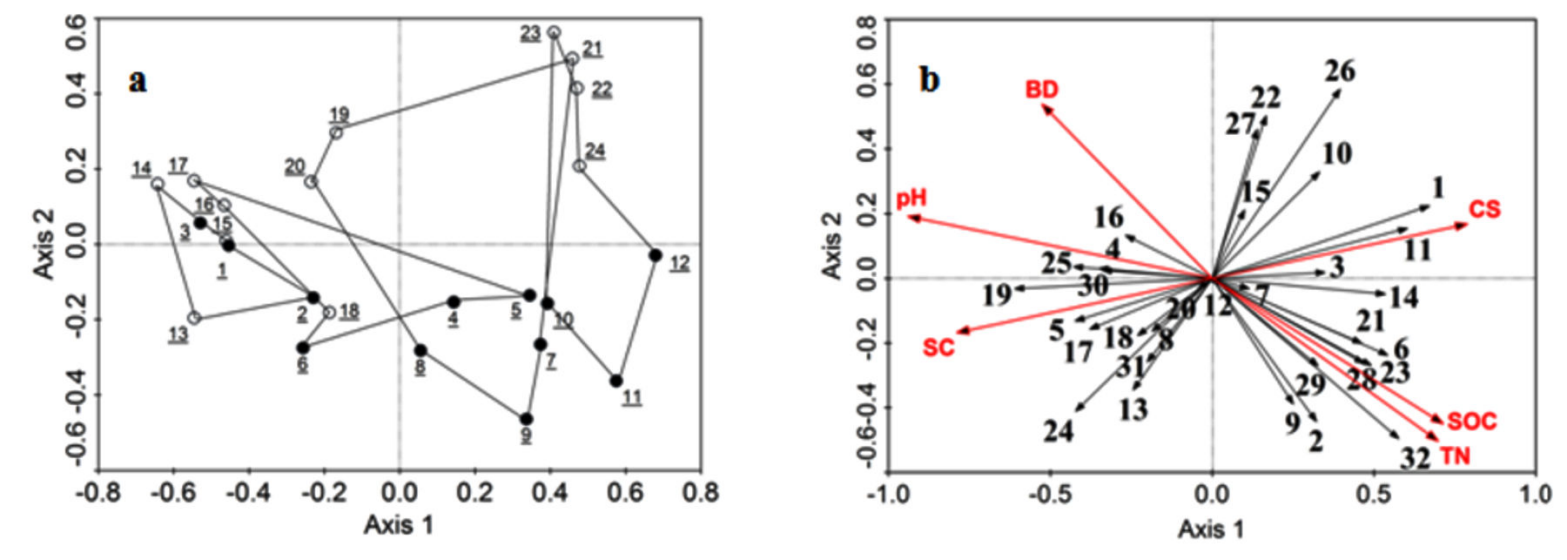
RDA two-dimensional ordination diagram of the first two axes showing the relationship between the community composition of ground arthropods and environmental variables.

Values are for Axes 1 and 2 plotted in the RDA diagram in Figure3. The highest canonical coefficients and correlations are highlighted by an asterisk (*) that indicates significance (* *p*<0.05). BD soil bulk density, CS soil clay plus silt content, pH soil pH, SC soil sand content, SOC soil organic carbon, TN total nitrogen.

Figure 3a shows the (dis)similarity between microhabitats among shrub plantation ages in terms of the abundance distribution of ground arthropods. Along axis 1 from left to right, 6 year-old plantations are separated from the other three ages (15, 24 and 36 years); in 6 year-old plantations, there is a high degree of similarity between the microhabitats, as evidenced by the crossing distributions of sampling microhabitats in the plot. From 15 to 36 years of plantation age, the microhabitat under shrubs (below axis 1) and in the open areas (above axis 1) is separated markedly from each other by axis 1; the differences between microhabitats increase drastically. Evidence indicated that the assemblage composition in the different microhabitats was strongly affected by plantation age (Figure 3a), which confirmed the previous results (Figures 1, 2), and mainly occurred in spring.

Figure 3b shows the distribution of arthropod groups in the RDA plot in terms of their abundances in relation to the environmental variables. The taxonomic group arrows that point in approximately the same direction as an environmental factor arrow indicate a strong positive correlation (the longer the arthropod group arrow, the stronger the relationship) [36,37]. Arrows for soil sand content, soil clay plus silt content and pH (first group of variables) were longer than those for soil bulk density, organic carbon and total nitrogen content (second group of variables), indicating that the first group of variables accounted for a greater proportion of the variance in arthropod community structure than the second. Soil pH, sand content and clay plus silt content had the strongest influences on the arthropod community, and these influences were significant (Table 2). The canonical coefficients and intra-set correlations for the environmental factors for each axis indicated that axis 1 primarily represented a gradient in soil pH, sand content and clay plus silt content (Figure 3a, b).

## Discussion

To our knowledge this is the first assessment of the influence of shrub plantation age on ground arthropod assemblages in different microhabitats across seasons. There were significant influences of microhabitats created by shrubs on total abundance, group richness and Shannon’s index as well as on the abundance distribution of such important groups such as Tenebrionidae and Formicidae (Table 1), as predicted. Results were in accordance with previous research on ground arthropod communities of shrub patches [3,9,11]. The positive effects of microhabitats on ground arthropods in this severely resource-limited desert steppe ecosystem could be ascribed to the multiple functions of shrubs, serving as primary producers, providers of resources and modulators of the physical environment (e.g., microclimate, soil nutrient and water availability) under their crown [10,39]. These ecological functions may combine to create a favorable microhabitat with relatively mild microclimates, secure oviposition sites, high resource availability and shelter, thereby resulting in a greater degree of arthropod activity and colonization in areas under shrubs versus those in open areas [3,6,39].

Interestingly, there was an interaction between microhabitat and plantation age on arthropod diversity (Table 1); as shrub plantation age increased, the difference in arthropod diversity between microhabitats tended to increase. This result implied that the influence of microhabitats on arthropod diversity was to some extent regulated by shrub plantation age [40]. As shrub age increases, the crown cover and number of shoots increase significantly (Figure S1) and some herbaceous vegetation invades the shrubs at early stages [18], which results in a more complex vegetation structure [14]. Large shrubs may attract abundant ground arthropods because these plants are the most conspicuous structural feature in the semi-natural sandy grasslands [33]. Ground arthropods may seek shrubs for thermal cover or may be attracted to the plant debris that accumulates under shrubs [41]. In addition, it is possible that the reduced mobility of the arthropods due to complex vegetation structure contributes to higher numbers of captures in traps under shrubs (higher probability of captures), because the movements of individual arthropods can be affected by the structural complexity of the surrounding vegetation [42].

However, the similarity of arthropod diversity between microhabitats in 36 year-old plantations indicated similar living conditions under shrubs to that in the open areas for ground arthropods. This pattern is consistent with what we know about relatively stable sandy grassland ecosystems [22,37]. When soil nutrients under shrubs expand into the open areas - causing soil conditions to improve and herbaceous vegetation to recover in the open areas as shrub age increase (Figure S2, S3) - there is an increasing attractiveness of the open areas to the ground arthropods [11,39]. When most arthropod groups then expand into the open areas, the arthropod diversity improve to levels similar to those under shrubs, thus enhancing the structure complexity of the soil food web and facilitating the recovery of degraded sandy grasslands [23,43].

In all, the total abundance, group richness and Shannon’s index, as well as all dominant groups were strongly affected by plantation age (Table 1). From 6 to 24 years of plantation age (Figures 1, 2), these community indices and most dominant groups (except for Formicidae) tended to decline, and after that they tended to increase, which contradicted our second prediction. There were lower values of these measurements in 15 and 24 year-old plantations, and particularly in the latter in comparison to the other plantation ages, which was perplexing and interesting. Previous studies showed that soil conditions and the cover of herbaceous vegetation improved to some extent (Figure S2, S3) [18] as shrub plantation age increased from 6 through 15 to 24 years of plantation age, displaying a positive relationship between plantation age and soil and vegetation properties [14,44]. However, to our knowledge, there is no information available as to why improved soil and vegetation are responsible for decreased community indices and the abundance of the most important groups. Historical grazing intensity may be a reasonable explanation for this result. The 15 and 24 year-old shrub lands had been continuously subjected to grazing pressure for the prior fifteen years, which was especially heavy for the latter plantation. Livestock grazing can negatively affect arthropods [45,46], and sufficiently heavy grazing pressure is expected to produce negative effects on arthropod assemblages even after the grazing has stopped [47]. A quantification of grazing pressure may be a key factor for soil amelioration and herbaceous vegetation recovery under post-grazing exclosure and this may be especially important in the open areas in this severely resource-limited desert steppe since it has been shown that the decomposition and mineralization of manure is an important source of nutrients when grazing is halted in the sandy grassland [46]. This relationship is intriguing and deserves more attention, especially for management of artificial plantations such as these semi-natural grassland ecosystems in arid regions.

In contrast, from 24 to 36 years of plantation age, most of dominant groups recovered and reached their peaks in total arthropod abundance, taxonomic richness, and Shannon’s diversity index (Figures 1, 2). Three observations may explain this pattern. Firstly, older (36 years) adult individuals of *C. koushinskii* are generally larger in size and above-ground biomass (Figure S1) than younger individuals, meaning that the older plantations contributed greater amounts of resources (leaves and litter) to arthropods and, more importantly, provided a greater number of potential oviposition sites for female arthropods [48]. Second, soil conditions such as soil bulk density, pH and organic carbon and total nitrogen improved markedly during the development of the shrub lands, not only under the shrubs but also in the open areas of these 36 year old plantations [18]. Third and most importantly, degraded grasslands tend to gradually stabilize and can be more resilient to disturbances from 24 to 36 years of plantation age, creating favorable conditions for most kinds of arthropods to burrow, lay eggs and survive [23,43].

As for Formicidae, there was a strong increase in abundance from 6 and 15 to 24 and 36 years of plantation (Figure 1). Perhaps richer herbaceous plants with abundant food resources and improved soil texture, soil organic carbon and total nitrogen [18,20] were strong attractants for this group in 24 and 36 year–old plantations [13]. In addition, different morphospecies of ants (most are herbivores), though not studied here, might require different age-related habitats in terms of food availability, vegetation and habitat quality [48]. Some morphospecies in the family Formicidae search for food primarily under shrubs and other understory plants [49,50], and therefore were predominant in older and larger old growth fragments such as 24 and 36 year–old plantations [51].

As predicted, significant effects of sampling period were observed on all community indices and dominant groups. Our results indicated that the distribution of ground arthropods in the desertified regions was dynamic in time (Figure 1, 2; Table 1). This result was in accordance with other studies [10,11,52], suggesting specific responses of ground arthropods to seasonal changes. Rainfall and temperature change with season, directly causing the abiotic changes that markedly influence the phenology of the arthropods and their feeding activities [11,52]. Phenology is an important biological attribute of arthropods, due to their long adaptations to local environmental conditions, though some groups are affected by the interaction of microhabitat (or plantation age) and season (Figure 1, Table 1). The phenology of arthropod groups due to birth/death dynamics and or diapause can provoke changes in abundance throughout the year [10]. For example, the Melolonthidae were found only in spring and summer (Figure 1). Seasonal changes such as drought and heavy rain may affect redistribution, survival and fecundity; seasonal temperature variations commonly induce vertical movements of soil/ground animals in the soil profile [53]. Indirectly, the phenology of plants shows a seasonal pattern of litter, root production and litter quality [3]. Moreover, the moisture content of the litter may affect the ability of juveniles to penetrate their substrates successfully [54]. These factors were potentially related to resource variation and availability [53]. For example, Carabidae predators were most abundant in the autumn; this peak in abundance coincided with peaks in abundance of numerous prey groups (such as herbivores) driven by the availability of food sources for the prey at that time [3].

In addition, the influences of microhabitats and plantation age were markedly affected by season (Table 1). In spring, higher measures of total abundance and group richness were observed under shrubs compared to those in the open areas, as mentioned above. However, the differences between microhabitats were reduced from spring to summer and autumn (Figure 2). In spring (shoot period), the microhabitats under the shrubs were the most attractive places with rich resources and suitable physical environments for these arthropod groups compared to those in the open areas [3,43]; most importantly, animals emerging from hibernation and hatching from eggs or larvae might also contribute to the arthropod community under the shrubs [34,35]; soil texture and conditions under the shrubs were very suitable for soil/ground arthropods to hibernate (in the form of eggs, larvae or adults) through the cold winter in comparison to those in the open areas [22]. However, after spring, the vegetation grew up with increases in rainfall and temperatures in the open areas; as a result, there were more similar living conditions between the microhabitats under shrubs and in the open areas, and the ease of mobility between microhabitats improved for ground arthropods [12,13]. The probability of captures in the open areas was as high as it was under shrubs, displaying the similarity of community indices between microhabitats in summer and spring.

Nevertheless, all these community indices were markably affected by the interaction between plantation age and season. From spring to summer, these community indices tended to decline from 6 to 24 years of shrub plantation age and after that they tended to increase strongly, as described above. Noticeably the lowest values in 24 year-old plantations occurred in summer, which may be related to the homogenization of vegetation structure [55]. During vegetation recovery in the growing period (summer) after heavy grazing, ground vegetation is mainly covered with mono-dominant plant species such as *Salsola collina* (vegetation cover above 65%), as observed in practice. Here, after summer there was a high degree of similarity in the indices measured, indicating only a slight effect of plantation age on community structure and diversity in autumn, a time when vegetation withers and the temperature decreases [3]. At this time, most arthropods have completed or are completing their life history, thus simplifying the community structure of arthropods and decreasing their diversity [53,56]. As a whole, from spring and summer to autumn, a similarity in these indices was observed among these four plantation ages, and total abundance, group richness and Shannon’s index declined markedly (Figure 2).

Using redundancy analysis (RDA), we assessed the relative importance of the measured environmental factors in structuring ground arthropod communities in the desertified steppe [36]. The RDA plot revealed that 6 year-old shrub plantations differed markedly from the others (Figure 3a); in 15, 24 and 36 year–old plantations, there were considerable differences between the microhabitats. This result supported findings discussed previously that showed the (dis)similarity among 6, 15, 24 and 36 year-old shrub plantations. The RDA also illustrated that the six environmental variables (i.e. soil texture, bulk density, pH, organic carbon and total nitrogen content) together explained 31.6% of the total variation in arthropod community composition. Soil pH, sand content and clay plus silt content had significant influences on the arthropod community (Figure 3b, Table 2). The canonical coefficients and intraset correlations for the environmental factors for each axis further indicated that the factors that most strongly influenced the distribution and composition of ground arthropods were soil pH, sand content and clay plus silt content. These results were consistent with other studies that suggested abiotic factors had important impacts, both directly and indirectly, on the distribution and diversity of ground arthropod communities [57,58]. This result also suggests that some other factors that were not considered in this study might also contribute to the unexplained variation [39].

In conclusion, a significantly higher distribution of arthropod assemblages was observed under the shrubs as compared to that in the open areas. The differences in arthropod communities between microhabitats were affected more by seasons rather than plantation age. We did not find a positive relationship between arthropod community diversity indices and plantation age, contradicting our second prediction, assuming that soil and/or vegetation quality would improve over time via re-vegetation by the shrubs after disturbances. Further, the influences of plantation age on arthropod communities were to some extent controlled by seasonal changes. The taxonomic group-, microhabitat- and season-specific signatures for ground arthropods were influenced by several key factors of abiotic conditions (historical or present), resulting in significant variation in community structure of ground arthropods between microhabitats, with shrub age and across seasons. Our understanding of the mechanisms that drove these changes would be improved by more detailed work on individual taxonomic groups of importance or functional groups [59], as well as by experiments testing the effects of abiotic conditions on arthropod behavior and biodiversity [39,60]. These findings have valuable implications for recovery managers, as they suggest varying responses of arthropods versus the soil and vegetation in recently revegetated grasslands. Consideration of these effects may be critical for successful conservation of shrubland-associated arthropod assemblage diversity and for recovery of desertified steppe ecosystems.

## Supporting Information

Figure S1 **Means (±SEs) of crown area, shrub height and aboveground biomass per shrub for each plantation age.**
(DOC)

Figure S2 **Means (±SEs) of soil properties between microhabitats for each plantation age.**
(DOC)

Figure S3 **Means (±SEs) of herbaceous characteristics between microhabitats for each plantation age averaged over three seasons (spring, summer, autumn).**
(DOC)

Table S1 **Mean abundance/traps (±standard error), as a function of microhabitat and shrub age in each season; zeros are omitted for clarity.**
(DOC)
