# Correction: Seasonal Distribution and Diversity of Ground Arthropods in Microhabitats Following a Shrub Plantation Age Sequence in Desertified Steppe

**DOI:** 10.1371/annotation/348b183b-9d62-4b07-bb09-b1ac6bcb9b09

**Published:** 2013-11-08

**Authors:** Rentao Liu, Fan Zhu, Naiping Song, Xinguo Yang, Yongqing Chai

There were multiple errors in the Funding Statement. The Funding Statement should read: "This project was supported by the National Natural Science Foundation of China (number 41101050) and National Science and Technology Support Program (2011BAC07B03). The funders had no role in study design, data collection and analysis, decision to publish, or preparation of the manuscript."

