# Supplementary material for: Seasonal Distribution and Diversity of Ground Arthropods in Microhabitats Following a Shrub Plantation Age Sequence in Desertified Steppe
Source: PLoS One. 2013 Oct 21;8(10):e77962. doi: 10.1371/journal.pone.0077962 (PMC3824025; doi:10.1371/journal.pone.0077962)
Supplement: Figure S1 — Means (±SEs) of crown area, shrub height and aboveground biomass per shrub for each plantation age. (DOC) [file pone.0077962.s001.doc]

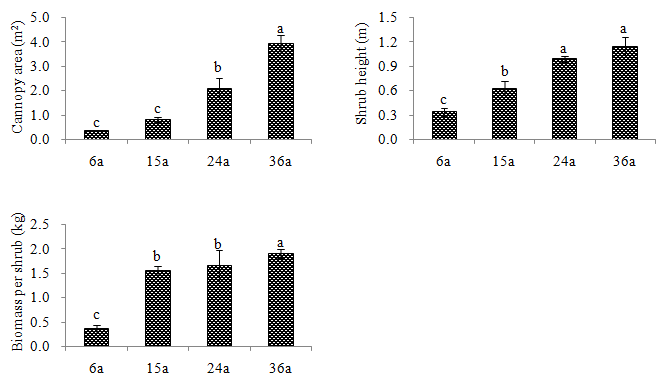


**Figure S1. Means (±SEs) of canopy area, shrub height and aboveground biomass per shrub for each plantation age. Different letters indicate significance ( *p* <0.05). (DOC)**
