# Supplementary material for: Seasonal Distribution and Diversity of Ground Arthropods in Microhabitats Following a Shrub Plantation Age Sequence in Desertified Steppe
Source: PLoS One. 2013 Oct 21;8(10):e77962. doi: 10.1371/journal.pone.0077962 (PMC3824025; doi:10.1371/journal.pone.0077962)
Supplement: Figure S2 — Means (±SEs) of soil properties between microhabitats for each plantation age. (DOC) [file pone.0077962.s002.doc]

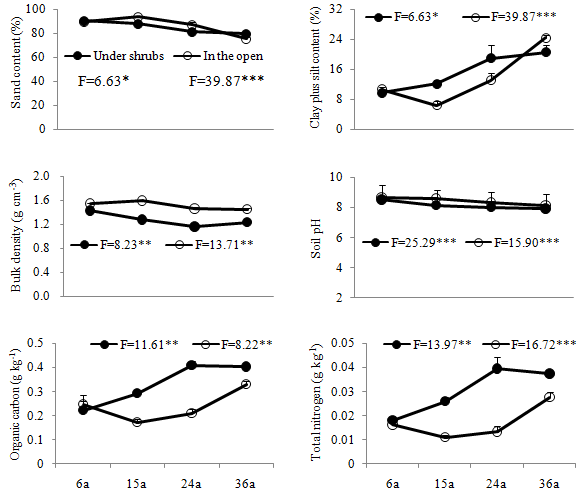


**Figure S2. Means (±SEs) of soil properties between microhabitats for each plantation age. Asterisk (*) indicates significance (**p* < 0.05, ***p* < 0.01, and ****p* < 0.001). (DOC)**
