# Supplementary material for: Seasonal Distribution and Diversity of Ground Arthropods in Microhabitats Following a Shrub Plantation Age Sequence in Desertified Steppe
Source: PLoS One. 2013 Oct 21;8(10):e77962. doi: 10.1371/journal.pone.0077962 (PMC3824025; doi:10.1371/journal.pone.0077962)
Supplement: Figure S3 — Means (±SEs) of herbaceous characteristics between microhabitats for each plantation age averaged over three seasons (spring, summer, autumn). (DOC) [file pone.0077962.s003.doc]

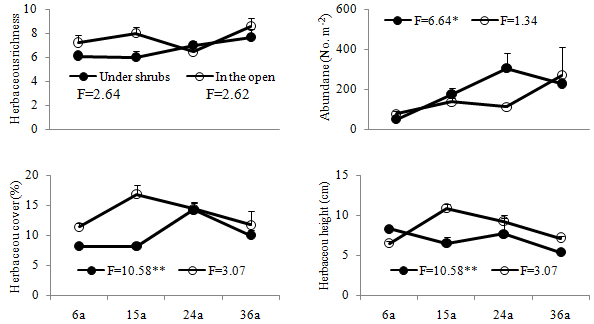


**Figure S3. Means (±SEs) of herbaceous characteristics between microhabitats for each plantation age averaged over three seasons (spring, summer, autumn). Asterisk (*) indicates significance (**p* < 0.05, ***p* < 0.01). (DOC)**
