# Supplementary material for: Seasonal Distribution and Diversity of Ground Arthropods in Microhabitats Following a Shrub Plantation Age Sequence in Desertified Steppe
Source: PLoS One. 2013 Oct 21;8(10):e77962. doi: 10.1371/journal.pone.0077962 (PMC3824025; doi:10.1371/journal.pone.0077962)
Supplement: Table S1 — Mean abundance/traps (±standard error), as a function of microhabitat and shrub age in each season; zeros are omitted for clarity. (DOC) [file pone.0077962.s004.doc]

**Supporting Information**

**Table S1. Mean abundance/traps (±SEs), as a function of microhabitat and shrub age in each season; zeros are omitted for clarity.** (DOC)

| **Spring** | | 6a | | 15a | | 24a | | 36a | |
| --- | --- | --- | --- | --- | --- | --- | --- | --- | --- |
| Order | Taxonomic group | Under shrubs | In the open | Under shrubs | In the open | Under shrubs | In the open | Under shrubs | In the open |
| Isopoda | Tylidae |  |  |  |  |  |  |  |  |
| Leiobunumecies | Phalangudae |  |  |  |  |  |  |  |  |
| Araneae | Lycosidae |  |  |  |  |  |  |  |  |
|  | Thomisidae |  |  |  |  |  |  |  |  |
|  | Gnapphosidae | 1.00±0.58 | 1.00±0.58 |  |  |  |  |  |  |
|  | Philodromidae |  |  |  |  |  |  | 2.67±1.20 | 1.00±1.00 |
|  | Liocranidae |  |  |  |  |  |  |  |  |
| Dermaptera | Labiduridae |  |  |  |  |  |  |  |  |
| Orthoptera | Arcypteridae |  |  |  |  |  |  |  |  |
| Hemiptera | Coreoidea |  |  |  |  |  |  |  |  |
|  | Scutelleridae |  |  |  |  |  |  |  | 1.00±0.58 |
|  | Lygaeidae |  |  |  |  |  |  |  |  |
| Coleoptera | Carabidae |  |  | 1.33±0.88 |  |  |  | 4.00±0.00 | 1.00±1.00 |
|  | Chrysomelidae | 1.00±0.58 |  |  |  |  |  | 21.00±11.59 | 3.67±1.76 |
|  | Buprestidae | 1.00±1.00 | 1.67±0.88 |  |  |  |  | 1.33±0.88 | 2.67±0.88 |
|  | Staphylinidae |  |  |  | 1.33±1.33 |  |  |  |  |
|  | Elateridae |  |  |  |  |  |  |  |  |
|  | Glaphyridae | 21.00±6.08 | 5.00±1.53 | 5.67±2.19 | 1.67±1.67 | 1.33±1.33 |  | 4.67±2.60 |  |
|  | Melolonthidae | 32.33±2.73 | 43.33±8.37 | 23.67±6.84 | 22.67±12.24 | 3.00±0.58 | 2.00±1.15 | 12.00±3.51 | 12.00±1.73 |
|  | Scarabaeidae | 4.00±2.31 | 7.67±7.17 |  |  | 2.33±1.86 |  | 1.33±0.88 | 1.33±0.33 |
|  | Aphodiidae |  |  |  |  | 1.67±0.67 | 1.33±1.33 | 2.00±1.00 | 1.00±1.00 |
|  | Silphidae |  | 1.00±1.00 |  |  |  |  |  | 3.33±1.67 |
|  | Histeridae |  |  |  |  |  |  | 1.67±0.88 |  |
|  | Teneberionidae adult | 60.00±12.00 | 47.00±2.08 | 20.00±4.51 | 3.00±2.08 | 19.67±5.49 | 7.33±4.33 | 28.67±2.60 | 11.67±1.20 |
|  | Curculionidae | 27.67±6.69 | 21.67±6.06 | 10.67±2.96 | 9.00±4.58 | 1.67±1.20 | 2.33±1.20 | 7.00±1.73 | 7.33±0.33 |
|  | Teneberionidae larvae |  |  |  |  |  |  |  |  |
| Diptera | Asilidae |  |  |  |  |  |  |  |  |
| Hymenoptera | Formicidae | 17.33±3.53 | 5.33±2.73 | 5.33±2.73 | 4.67±4.18 | 48.67±19.46 | 21.33±15.72 | 84.00±24.00 | 36.00±6.53 |
|  | Megachilidae |  |  |  |  |  |  | 1.00±0.58 |  |
|  | Scoliidae |  |  |  |  |  |  |  |  |
|  | Sphecidae |  |  |  |  |  |  |  |  |
| Lepidoptera | Larvae |  |  | 5.33±3.38 |  | 1.67±0.33 |  | 9.00±4.04 |  |

| **Summer** | 6a | | 15a | | 24a | | 36a | |
| --- | --- | --- | --- | --- | --- | --- | --- | --- |
|  | Under shrubs | In the open | Under shrubs | In the open | Under shrubs | In the open | Under shrubs | In the open |
|  |  |  |  |  |  |  |  |  |
|  |  |  |  |  |  |  | 1.00±0.58 |  |
|  |  |  |  |  |  |  |  | 1.00±0.58 |
|  |  |  |  |  |  |  |  |  |
|  |  |  |  |  |  |  |  |  |
|  |  |  |  |  |  |  |  |  |
|  |  |  | 1.00±1.00 | 1.33±0.88 |  |  |  | 1.00±0.58 |
|  |  | 1.67±0.33 |  |  |  |  | 1.00±0.58 |  |
|  |  |  |  |  |  |  |  |  |
|  |  |  |  |  |  |  |  | 1.00±0.58 |
|  |  |  |  |  |  |  | 2.67±1.76 | 1.33±1.33 |
|  |  |  |  | 3.67±3.67 |  |  | 1.67±0.88 |  |
|  |  | 2.00±0.00 | 3.33±0.33 | 2.33±0.33 | 1.33±0.67 |  | 6.00±2.08 | 1.67±1.20 |
|  |  |  |  |  |  |  |  |  |
|  |  |  |  |  |  |  |  |  |
|  |  |  |  |  |  |  |  |  |
|  | 3.00±1.00 |  | 1.33±1.33 |  |  |  |  |  |
|  | 2.33±1.86 |  |  |  |  |  |  | 1.67±0.67 |
|  | 12.67±4.33 | 9.67±1.45 | 5.33±2.60 | 13.33±2.91 | 1.00±0.58 | 3.00±1.00 | 5.67±1.76 | 4.67±1.76 |
|  |  |  |  |  |  |  |  |  |
|  |  |  |  |  |  |  |  |  |
|  |  |  |  |  |  |  |  |  |
|  |  |  |  |  |  |  | 1.00±0.58 |  |
|  | 16.33±4.41 | 8.33±1.20 | 8.67±2.85 | 5.33±0.67 | 13.00±6.56 | 1.33±1.33 | 13.67±3.18 | 4.00±0.58 |
|  | 2.00±1.15 |  |  |  |  |  | 2.00±2.00 | 1.00±0.58 |
|  |  |  |  |  |  |  |  |  |
|  |  |  |  |  |  |  |  |  |
|  | 8.33±3.84 | 8.33±4.84 | 8.00±6.56 | 20.67±10.48 | 1.67±1.20 |  | 4.00±2.08 | 6.67±3.71 |
|  |  |  |  |  |  |  |  |  |
|  |  |  |  |  |  |  |  |  |
|  |  |  | 1.67±0.33 |  |  |  |  |  |
|  |  |  |  |  |  |  |  |  |

| **Autumn** | 6a | | 15a | | 24a | | 36a | |
| --- | --- | --- | --- | --- | --- | --- | --- | --- |
|  | Under shrubs | In the open | Under shrubs | In the open | Under shrubs | In the open | Under shrubs | In the open |
|  |  |  |  |  |  |  | 2.33±0.67 | 3.00±1.00 |
|  |  |  | 3.67±1.20 |  | 1.00±1.00 |  |  |  |
|  |  |  |  |  | 1.00±1.00 |  |  |  |
|  | 1.33±0.67 |  |  |  |  |  |  |  |
|  |  |  |  |  |  |  |  |  |
|  |  |  | 1.33±0.33 |  |  |  | 1.00±0.58 |  |
|  |  |  |  | 1.00±0.58 |  |  |  |  |
|  |  |  |  |  |  |  |  |  |
|  |  |  |  |  | 1.00±0.58 |  |  |  |
|  |  |  |  |  |  |  |  |  |
|  |  |  |  |  |  |  |  |  |
|  |  |  |  |  |  |  |  |  |
|  | 48.00±29.14 | 27.00±7.94 | 12.33±1.33 | 8.33±1.76 | 9.33±9.33 | 1.00±0.58 | 14.00±11.50 | 1.00±0.58 |
|  |  |  |  |  |  |  |  |  |
|  |  |  |  |  |  |  |  |  |
|  |  |  |  |  |  |  |  |  |
|  |  |  |  |  |  |  |  |  |
|  |  |  |  |  |  |  |  |  |
|  |  |  |  |  |  |  |  |  |
|  |  |  | 1.00±1.00 |  |  |  | 1.00±1.00 |  |
|  |  |  |  |  |  |  |  |  |
|  |  |  |  |  |  |  |  |  |
|  |  |  |  |  |  |  |  |  |
|  | 8.33±2.60 | 4.33±2.03 | 2.33±0.88 | 15.00±0.58 | 7.00±4.58 | 8.67±5.24 |  | 2.00±1.00 |
|  |  |  |  |  |  |  |  | 1.33±0.67 |
|  |  |  |  |  |  |  |  | 1.67±0.33 |
|  |  |  |  |  |  | 1.00±0.58 |  |  |
|  | 15.67±8.67 | 6.67±3.76 | 5.67±1.20 | 9.67±0.33 | 3.33±0.88 | 6.67±2.03 | 16.67±2.60 | 6.67±0.33 |
|  |  |  |  |  |  |  |  |  |
|  | 1.00±0.58 |  |  |  |  |  |  |  |
|  | 1.00±0.00 |  |  |  |  |  |  |  |
|  |  |  |  |  |  |  |  |  |
